# Editorial: Innovative therapeutic strategies for managing diabetic foot ulcers and mitigating associated complications

**DOI:** 10.3389/fphar.2026.1788742

**Published:** 2026-03-19

**Authors:** Calvin A. Omolo, Vinod Kumar Yata, Yasodha Krishna Janapati, Sudharshan Reddy Dachani

**Affiliations:** 1 School of Pharmacy and Health Sciences, United States International University - Africa, Nairobi, Kenya; 2 Discipline of Pharmaceutical Sciences, College of Health Sciences, University of KwaZulu-Natal, Durban, South Africa; 3 Department of Biotechnology, School of Allied and Healthcare Sciences, Malla Reddy University, Hyderabad, Telangana, India; 4 Department of Pharmacology and Clinical Pharmacy, College of Pharmacy, Shaqra University, Shaqra, Saudi Arabia

**Keywords:** advanced drug delivery systems, chronic wound healing, diabetic foot ulcers, polyherbal and integrative therapies, precision medicine, wound microbiome

## Introduction

Diabetic foot ulcers (DFUs) represent one of the most severe, costly, and debilitating complications of diabetes mellitus, evolving into a critical public health challenge of global proportions. The statistics show that over half a billion adults live with diabetes worldwide, and approximately 6.3% will develop a foot ulcer in their lifetime (Wang et al.). This translates to an estimated 18.6 million new ulcers annually (Armstrong et al., 2023). The consequences are devastating, with a DFU-related amputation occurring somewhere in the world every 20 s (Li et al., 2023). The 5-year mortality rate following ulceration rivals that of many diseases such as cancers, underscoring the life-threatening nature of this condition.

The pathophysiology of DFUs is a complex interplay of neuropathy, peripheral arterial disease, and a profoundly impaired immune and healing response. This complexity means that conventional therapies centred on debridement, infection control, and offloading often fall short, leading to high recurrence rates and progressive tissue loss (Aditya et al.). The diabetic pandemic is projected to affect 783 million people by 2045 (Hossain et al., 2024). This demands a paradigm shift from reactive wound management to proactive, innovative, and holistic healing strategies.

It is with this reason the Frontiers Research Topic, *“Innovative Therapeutic Strategies for Managing Diabetic Foot Ulcers and Mitigating Associated Complications,”* seeks to address. This compendium of 22 articles provides a comprehensive snapshot of the cutting-edge science driving the field forward. This editorial moves beyond a simple aggregation of findings to critically evaluate how this Research Topic of studies collectively challenges and expands the current DFU management paradigm. We highlight where convergence occurs, identify persistent evidence gaps, and argue for a prioritized research agenda focused on translatable, equitable solutions.

## Novel pharmacological interventions

Moving beyond broad-spectrum antibiotics, contemporary research focuses on targeted, intelligent strategies and agents that address the underlying pathophysiology of chronic wounds, which is paramount in tackling DFU. Strategies presented in this Research Topic include precision antibiotic therapy. The work by Zhang et al. exemplifies this shift, employing a Boruta algorithm to analyze the wound microbiome and guide the selection of antibiotics for loading into bone cement. This data-driven approach aimed to combat multidrug-resistant infections more effectively than empirical treatment (Zhang et al.). Studies reported also explored targeting specific pathological mechanisms in DFU. Li et al. unveiled cuproptosis as a potential new therapeutic target, highlighting a novel mechanistic pathway; however, its clinical relevance and therapeutic manipulability remain to be established (Li et al.). While these targeted strategies represent a significant leap from empirical treatment, their clinical readiness varies. Algorithm-guided therapy faces hurdles in real-time microbiome diagnostics and accessibility outside specialized centers. Cuproptosis, though a mechanistically fascinating target, requires extensive preclinical validation to assess therapeutic safety and efficacy before clinical trials can be considered.

## Polyherbal formulations and natural remedies

The therapeutic potential of plant-based medicine, rooted in centuries of traditional knowledge, is being rigorously validated by modern science. These polyherbal approaches often offer multi-target synergy, such as addressing inflammation, oxidation, and infection simultaneously. The review by Feng et al. provided a crucial overview, examining not only the efficacy of various polyherbal formulations through novel delivery systems (like nanoparticles and hydrogels). This formulations are being designed to enhance the bioavailability and targeted release of their bioactive components. A historical lack of mechanistic insights has hindered the application of natural remedies in DFU. Contemporary research that prioritizes elucidating the molecular pathways in DFU such as modulation of NF-κB, Nrf2, or growth factor signaling, was reported through which herbs like *Curcuma longa*, *Aloe vera*, and *Centella asiatica* which promotes healing (Feng et al.; Wang et al.). Despite this promising research, major challenges impede the integration of polyherbal therapies into mainstream DFU care. These include a lack of standardized extracts with consistent potency, limited large-scale randomized controlled trials assessing hard endpoints like amputation prevention, and complex regulatory pathways for multi-compound botanical products. Future work must address these issues to translate traditional wisdom into evidence-based practice.

## Advanced drug delivery systems

Innovative biomaterials are revolutionizing wound care by creating a protective, bioactive environment that actively guides the healing process. Such biomaterials include Hyaluronic acid (HA), which has been employed to develop various advanced drug delivery systems for DFU treatment. The systematic review and meta-analysis by Yao et al. consolidated evidence on the effectiveness of HA and its derivatives, which maintain a moist wound environment, facilitate cellular migration, and can be engineered for controlled release of drugs or growth factors (Yao et al.). Original research, such as the study by Wang et al. on negative pressure wound therapy (NPWT) combined with silver-ion dressings, demonstrated how advanced delivery systems can work in concert to reduce pro-inflammatory cytokines (IL-6, TNF-α) and improve healing outcomes through a combination of dressings (Wang et al.). However, the clinical translation of such sophisticated biomaterial systems faces significant hurdles, including high costs, regulatory complexities for combination products, and the need for robust comparative effectiveness trials against standard care in diverse healthcare settings.

## Wound microbiome modulation

Recognizing the DFU as a distinct ecological niche, research is shifting from simply eradicating infection to understanding and modulating the entire microbial community. The review by Wang et al. delves into the mechanisms of microbial infection across healing phases, emphasizing how dysbiosis (an imbalance in the microbial community) contributes to chronicity. The aforementioned study by Zhang et al. on algorithm-guided antibiotic selection is a direct clinical application of microbiome science, aiming to suppress pathogenic bacteria while preserving a healthier microbial balance.

## Clinical translation and personalized medicine

Bridging the gap between laboratory innovation and patient bedside requires robust clinical trial frameworks and tools for individualized risk assessment. The methodological scoping review by Zhang et al. critically examined the design and reporting of DFU clinical trials, providing essential guidance for generating higher-quality, more generalizable evidence (Zhang et al.). Personalization begins with prevention. Zhang et al. developed and validated a nomogram to predict the risk of moderate-to-severe DFUs in patients with type 2 diabetes, enabling earlier, targeted interventions (Zhang et al.). Therefore, strengthening the foundational evidence through rigorous trial design and directly translating that evidence into actionable, patient-specific prognostic tools together form a powerful strategy for advancing the prevention and management of diabetic foot ulcers.

## Considerations for global implementation and clinical translation

The promising strategies outlined herein must be scrutinized through the lens of global health equity. Advanced biomaterial dressings, microbiome sequencing, and complex polyherbal formulations may be cost-prohibitive or infrastructurally untenable in low- and middle-income countries (LMICs), which bear a significant portion of the DFU burden. Future innovation must, therefore, parallel the development of simplified, affordable, and robust adaptations, such as low-cost point-of-care diagnostic tools, locally sourced, standardized herbal products, and task-shifted care models. This is to ensure these advances do not widen existing health disparities.

## Conclusion

The work in this Research Topic signals a shift from a one-size-fits-all model to a multipronged, precision-based approach to DFU. The novelty lies in the integrated vision the authors present by leveraging computational tools (like microbiome analysis), advanced engineering (in biomaterials), and traditional knowledge simultaneously. To realize this vision, future research must prioritize the need to combine technology and tradition by merging advanced biomaterials and devices with the validated wisdom of polyherbal and integrative medicine. Furthermore, this integrated approach must bridge mechanism and translation, ensuring novel discoveries are grounded in a deep understanding of pathophysiology and rigorously tested in well-designed clinical trials. Collectively, these studies highlight a translational gap between innovative discovery and routine practice. Key barriers include the high cost of advanced biomaterials, the need for standardized protocols in polyherbal medicine, and the lack of large-scale, pragmatic trials for personalized risk prediction tools. Ultimately, all innovation must be directed toward holistic and accessible care. There is a need for designing treatment strategies that are feasible and scalable across diverse global healthcare settings. The burden of DFUs is too great, and the human cost too high, to tolerate fragmented approaches. We call for increased collaborative research, cross-disciplinary dialogue, and investment in healthcare systems that can deliver these innovative, integrated strategies. By doing so, we can transform the forecast for millions of patients, turning a trajectory of amputation and mortality into one of healing, restored function, and hope.
